# Current methods in translational cancer research

**DOI:** 10.1007/s10555-020-09931-5

**Published:** 2020-09-14

**Authors:** Michael W. Lee, Mihailo Miljanic, Todd Triplett, Craig Ramirez, Kyaw L. Aung, S. Gail Eckhardt, Anna Capasso

**Affiliations:** 1 Department of Medical Education, Dell Medical School, University of Texas at Austin, Austin, TX USA; 2 Department of Oncology, Dell Medical School, University of Texas at Austin, Austin, TX USA; 3 Livestrong Cancer Institutes, Dell Medical School, University of Texas at Austin, Austin, TX USA

**Keywords:** Translational research, Cancer, GEMMs, PDX, Xenograft, tumor immunology

## Abstract

Recent developments in pre-clinical screening tools, that more reliably predict the clinical effects and adverse events of candidate therapeutic agents, has ushered in a new era of drug development and screening. However, given the rapid pace with which these models have emerged, the individual merits of these translational research tools warrant careful evaluation in order to furnish clinical researchers with appropriate information to conduct pre-clinical screening in an accelerated and rational manner. This review assesses the predictive utility of both well-established and emerging pre-clinical methods in terms of their suitability as a screening platform for treatment response, ability to represent pharmacodynamic and pharmacokinetic drug properties, and lastly debates the translational limitations and benefits of these models. To this end, we will describe the current literature on cell culture, organoids, *in vivo* mouse models, and *in silico* computational approaches. Particular focus will be devoted to discussing gaps and unmet needs in the literature as well as current advancements and innovations achieved in the field, such as co-clinical trials and future avenues for refinement.

## Introduction

Extensive efforts directed towards mapping the cancer genome have yielded remarkable insight into the genomic changes that occur during tumorigenesis. Analysis of 2658 whole-cancer genomes from 38 tumor types by the Pan-Cancer Analysis of Whole Genomes (PCAWG), Consortium of the International Cancer Genome Consortium (ICGC), and The Cancer Genome Atlas (TCGA) demonstrated that on average, cancer genomes contain 4–5 driver mutations from coding and non-coding genome elements [1]. They also found that approximately 5% of tumors had no identifiable driver, suggesting that additional unidentified driver genes exist [1]. In one of several companion articles published by the ICGC/TCGA/PCAWG, an evolutionary history of the cancers based on the aforementioned sequence data was identified [2]. Based on these data, it is not far-fetched to conceive that rational deployment of therapeutics or interventions could shift the evolutionary trajectory of the malignant phenotype.

When taken together, the voluminous amount of cancer genome sequencing data that has been generated provides a high-fidelity roadmap of tumorigenesis that can be experimentally exploited to develop a predictive, formulaic method of treating cancer based on mutational changes. But understanding the biological processes of cancer progression is only part of the equation. Knowledge of how the tumor genome changes and adapts upon exposure to therapeutic agents and environmental carcinogens is equally important, as is deciphering the role of epigenetics and protein modifications in oncogenesis. Thus, it will be the role of translational research tools to unravel the complexities of treatment response and resistance, and how this alters the trajectory of tumor development and progression, in the face of genomic changes.

There is an imperative to develop a multi-faceted approach towards modeling cancers in the laboratory that are eminently translatable into the clinical environment. However, experimentally modelling tumor development so that it provides accurate, clinically meaningful, and actionable data to screen patients for risk, treatment selection, and prediction of treatment adverse effects is challenging, in part because tumors are heterogeneous entities.

Tumor heterogeneity and clonal evolution pose formidable barriers to studying cancer biology, immune-tumor interactions, and the response of cancers to therapeutic agents [3–6]. Variations exist between different cancer types in terms of their genetic and epigenetic heterogeneity and, furthermore, clonal evolution can be altered by exposure to chemotherapeutics [6–8]. Tumor heterogeneity is not restricted to the tumor and can extend into the tumor microenvironment, including non-tumor fibroblasts, immune cells, endothelial cells, and matrix components, that can influence propagation of a tumor and its response to therapy [9].

Therefore, translational models for studying cancer should, ideally, provide an environment for cancers to progress along their natural course of evolution so that tumor heterogeneity can be studied in the presence and absence of therapeutics.

Here, we will explore the current literature covering *in vitro* tools such as traditional cell line based tissue culture and newer *in vitro* methods such as 3D organoid models that more accurately simulate the *in vivo* tumor environment. *In vivo* modalities such as xenografts and syngeneic mice, genetically engineered mice, and patient-derived xenografts will also be discussed. Lastly, *in silico* methods will be reviewed with a focus on bioinformatics and computational tools that can be used to model tumor evolution and drug sensitivity. As will be discussed, some of these tools are more aptly suited for exploring tumor evolution and heterogeneity, whereas others are more relevant for studying metastasis, drug discovery, or screening novel compounds.

## Cell culture

**Fig. 1:**
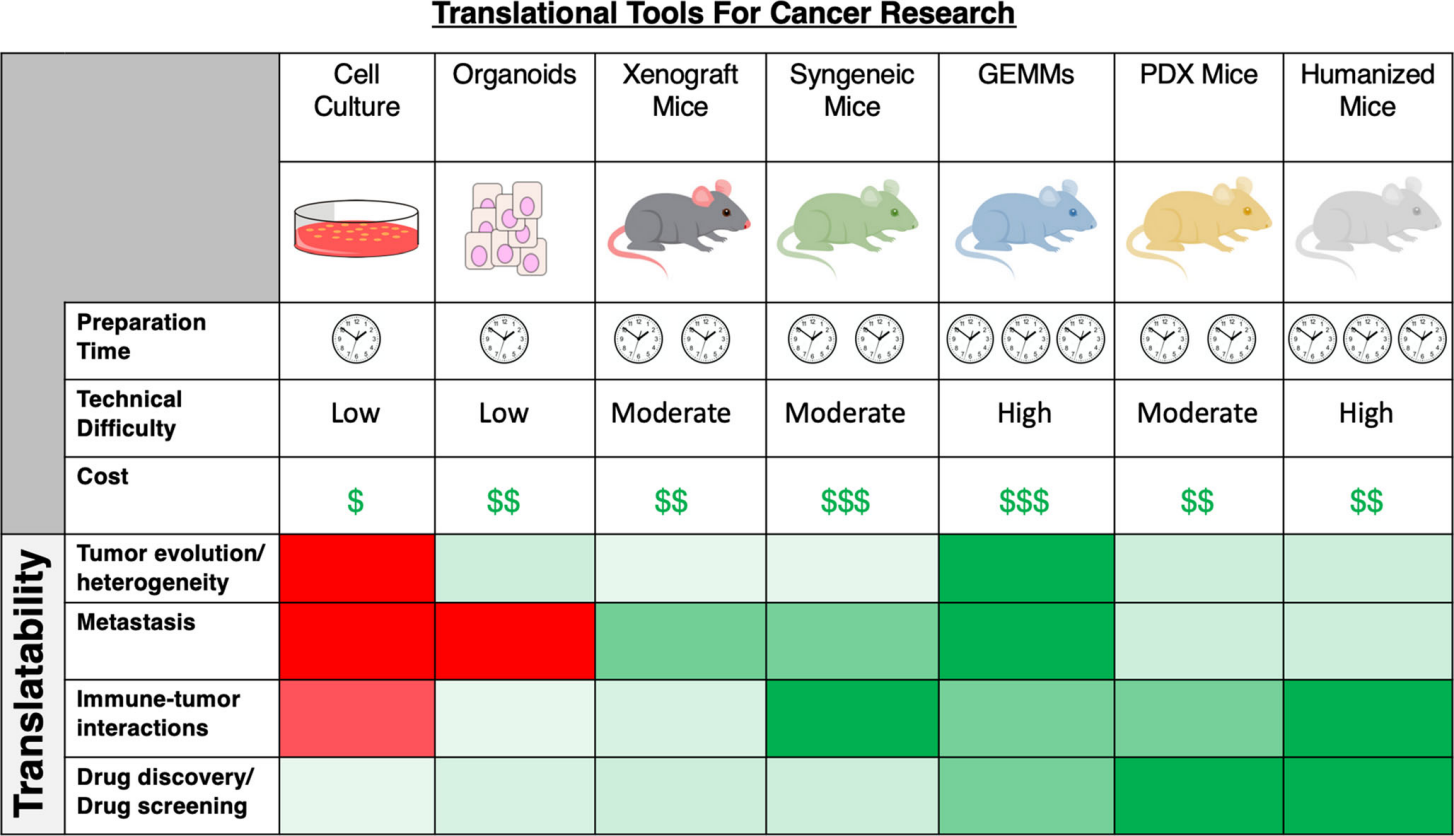
Comparison of strengths and weaknesses of current models for translational cancer research**.** For each model, the number of clocks and dollar signs correspond to the preparation time and relative cost of establishing and maintaining the model. Likewise, color coding indicates the degree to which the model is suited for a particular type of translational research (dark red denoting poorly suited to dark green denoting well suited)

Cell culture has long been a platform to discover gene alterations in cancer, identify aberrant signaling pathways, and screen new chemical entities as potential chemotherapeutic agents (Fig. 1). While there are many drawbacks and well-known shortcomings of traditional cell culture such as a lack of three dimensional architecture, changes in drug responsiveness, and growth changes with repeated passage, as well as limitations in studying drug metabolism and metastasis; the majority of current knowledge about the biology of cancer cells has been discovered using cell culture. Numerous studies have shown that cell culture systems can model genomic and transcriptomic changes seen in primary tumors [10, 11]. For example, Barrentina et al. used DNA copy number and gene expression patterns to determine equivalency between a human cell line library, with 947 cell lines, and primary tumors from corresponding tissues [11]. They reported positive correlations for DNA copy number (*r* = 0.77), gene expression patterns (*r* = 0.60), and point mutation frequency (*r* = 0.71) for all but a small number of the cell lines examined [11]. On the basis of the gene expression profiles, subsequent pharmacological interrogation of this cell line library revealed several previously unrecognized genes and cell features that correlate with drug response. For example, expression of the aryl hydrocarbon receptor (AHR) was found to correlate with enhanced response to MEK inhibitors in NRAS mutant cell lines [11].

However, a central issue with cell culture systems is that, although driver mutations are generally preserved, prolonged culturing can lead to secondary genomic changes including copy number variations and transcriptomic drifts [12, 13]. Indeed, these types of culture-condition-induced alterations can lead to changes in multi-drug resistance genes that differ from clinical samples. This observation was first made while evaluating over 80 samples of untreated primary ovarian carcinoma in comparison with additional cancer types from the NCI-60 panel and evaluating the expression profile of over 380 MDR-related genes [7]. These authors expanded their analysis to include several other cancer types including colorectal cancer, breast cancer, metastatic melanoma, and glioblastoma, which similarly demonstrated that cultured cell pairs from a primary tumor bore more resemblance to each other than pairs from different primary tumors of the same origin [7]. This indicates that cultured cells can retain genomic signatures from the primary tumor despite the influence of *in vitro* culture conditions.

In addition to the effects of prolonged culture conditions, there are also other drawbacks of traditional 2D cell systems. For example, the absence of extracellular architecture, including stromal cells and matrix components, can alter innate biological processes of cultured cancer cells and modify their response to therapeutics [14]. It has been shown that inclusion of tumor stromal cells and extracellular matrix mediators can drive tumor growth, stimulate angiogenesis, favor an inflammatory environment, and promote drug resistance [10, 14–16]. For these and other reasons, 3D tissue culture and organoid systems were developed.

There are a number of studies that have demonstrated the impact that incorporating stromal cells and matrix components can have on the response of cancer cells to therapeutics [17–19]. In some cases, this involved developing novel culture systems. For example, a recently published study described the development of a 3D tumor invasion model that utilizes traditional cell culture together with a customized system that incorporates extracellular matrix (ECM) [20]. In this study, the authors used highly metastatic pancreatic ductal adenocarcinoma (PDAC) cells as their model. Briefly, a fabricated platform was developed with posts coated in ECM and arranged in a 96-well format upon which tumor cells suspended in type I collagen oligomer were seeded. Following initial polymerization of the oligomer, a plug-like tumor compartment at the bottom of the 96-well plate formed onto which media, drugs, and cancer-associated fibroblasts (CAFs) could be overlaid. They noted that the addition of the CAFs resulted in dramatic changes in the phenotype of both PDAC cells and CAFs together with matrix remodeling and, importantly, pronounced invasion into the surrounding matrix [20]. Markers of epithelial to mesenchymal transition (EMT) were also examined and suggested an EMT-independent invasion phenotype [20]. A proof-of-concept drug screening treatment regimen with 10 different doses of gemcitabine was performed using Hoechst 33342, Click-iT EdU, and Mito Tracker Red (to assess nuclear changes/condensation, proliferation, and mitochondrial metabolism, respectively). Their results revealed effects of gemcitabine on cell proliferation, although with only moderate effects on invasion. Based on these data, the authors were able to establish initial validation of this system as a potentially viable drug screening platform. They also noted that this system has distinct advantages over other established models of migration/invasion/metastasis such as scratch assays, transwell (Boyden chambers), and 3D spheroid invasion assays in terms of standardization of spheroid and matrix components for high throughput/high content screening [20].

Another drawback of using cell culture as a clinically predictive model is the minimal degree of drug metabolism that occurs in a single-cell lineage context. Drug metabolism, particularly cytochrome P450–mediated drug metabolism, may yield a mixture of active and inactive metabolites that ultimately contributes to the pharmacological efficacy of chemotherapeutic agents under investigation [21]. This complexity of metabolism is lost in cell culture systems. Furthermore, the lack of a normal control for tissue culture is an important factor [22].

Although isogenic cell lines can be generated using homologous recombination to provide a comparator cell line when analyzing single gene differences, this technique is still hampered by cell culture effects [21]. Studies have demonstrated that there can be notable differences in cell expansion and drug sensitivity between identical isogenic cell lines as a result of 2D or 3D conditions [23]. For example, the isogenic DLD1 *KRAS*^+/−^, *KRAS*^G13D/−^, *PIK3CA*^+/−^, and *PIK3CA*^E545K/−^ colorectal cancer cell lines exhibited substantially different growth kinetics and sensitivity to the MEK inhibitor PD 0325901 depending on whether cells were cultured in 2D or 3D conditions despite their identical, isogenic status [23]. Due to these limitations, other methods of tissue culture have emerged that more closely recapitulate clinical heterogeneity while limiting artificial cell culture effects [22].

## Organoids

Organoids are an advancement of traditional tissue culture that is meant to more closely mimic the 3D architecture of primary tumors. Hans Clevers defined an organoid as “a 3D structure grown from stem cells and consisting of organ-specific cell types that self-organize through cell sorting and spatially restricted lineage commitment” [24]. As noted in this conventional definition, organoids can be derived from embryonic, adult, or pluripotent stem cells [25]. However, somatic cells can also be conditionally reprogrammed and cultured as organoids using feeder cells and Ras homolog (RHO) kinase inhibitors [25]. This method is not to be confused with somatic cell reprogramming which involves generation of induced pluripotent stem cell (iPSCs) from somatic cells using techniques such as somatic cell nuclear transfer (SCNT), cell-cell fusion, exposure to extracts of pluripotent cells, or iPSC technology [26]. It has been previously shown that organoids may be established from a variety of tumor types, such as colon, pancreas, esophageal, liver, endometrial, breast, and prostate cancers all requiring different composition of cultural media [27]. Previous studies have shown that organoids can maintain similar histopathological features derived from the primary tumor not only in the *in vitro* setting but also after being injected into immunocompromised mice permitting their use as an efficient tool to validate drug responses obtained *in vitro* and also in more complex *in vivo* systems [27]. In fact, their ability to better recapitulate tumor structure may have a greater impact on predicting responses to novel and conventional anti-cancer therapeutics with respect to 2D cell lines, opening an avenue towards drug development and personalized medicine [27]. Organoids are not without downsides, however. As will be discussed, they are slightly more technically and time intensive than traditional cell culture, can be subject to overgrowth and passage effects, and have limitations similar to cell culture relating to drug metabolism.

In general, organoids have a number of important features that set them apart from traditional cell culture and animal models [22, 28]. They self-organize and mimic the general architecture of the tissue of origin, and, importantly, maintain these characteristics over successive passages. This more relevant *in vitro* model offers advantages for studying tumor progression, treatment responsiveness, and interactions with the immune system and the tumor microenvironment (Fig. 1). Moreover, the morphological stability of organoids allows them to be coupled with other powerful techniques such as CRISPR/Cas9 and single-cell analysis [28]. As will be discussed below, organoids are also genetically stable models [24, 29].

The most common method of generating organoids from normal and tumor tissue is with adult stems cells isolated from resected tissue or biopsies using conditioned media supplemented with growth and selection factors [24, 25]. Most organoid media is supplemented with R-spondin, Wnt, epidermal growth factor (EGF), and Noggin, together with the ALK (anaplastic lymphoma kinase) inhibitor A83-01, p38 inhibitor SB202190, and nicotinamide [28]. Lgr5 is a G protein–coupled receptor that is found on stem cells and binds R-spondin, whereas Wnt (i.e., Wnt-3A) is a ligand for Frizzled receptors found on Lgr5^+^ stem cells [28]. Noggin is included because it is a bone morphogenic protein (BMP) receptor inhibitor. BMP receptor engagement on Lgr5^+^ stem cells negatively regulates stemness, whereas EGF binding to EGF receptors on Lgr5^+^ stem cells increase stemness [28]. A83-01 and SB202190 both appear to increase the number of passages of organoids and their long-term culture [29]. This method of culturing adult stem cells has been validated in a variety of tissue and tumor types although important differences exist between tissues in terms of specific composition of the growth media [24]. In addition to distinct culture requirements, organoid culture success rates can vary significantly between different cancer types [30].

As noted, adult stem cell–derived organoids are more frequently employed and appear to have a number of advantages over pluripotent stem cell organoids in terms of retention of phenotypic tissue features, biobanking, genetic modification, generation of matched normal controls, and incorporation of an immune system, among others [22].

However, a central question to consider with organoids, given the dynamic and heterogeneous nature of the cancer genome, is how well do organoids reflect the genetic and mutational profile of the parent tumor and how stable are the genetics over the study/treatment period? In other words, do the conditioned and semi-artificial culture conditions of organoid growth environments result in deviation of tumors from their inherent genetic mutational evolution?

### Modeling tumorigenesis and tumor evolution

As noted, intensified focus on the evolution and development of tumors has created a need to craft systems capable of modeling these processes [28]. Organoids appear to be a flexible system in terms of genetic manipulation and therefore can be used as a platform for discovery of novel genes involved in tumorigenesis [31]. Building on a previous study that used a *Sleeping Beauty* transposon–based mutagenesis screening system, Takeda et al. used a CRISPR-Cas9 gene-editing strategy to knockout genes in intestinal organoids derived from both mice and from human colorectal tumors [32, 33]. This system has significant advantages over knockout mouse models in terms of assessing cancer driver gene function, particularly in terms of cost and time. From a pool of genes they identified, they selected 29 candidate tumor suppressor genes (including *Trp53*, *Smad4*, and *Pten*) for loss-of-function studies. Using organoids sourced from mouse intestinal tumors with a mutated APC and mutated Kras background (APC^Δ716^, Kras ^+/G12D^) (both commonly mutated genes in colorectal cancer), they transduced the organoids with lentiviral vectors containing Cas9 and GFP, followed by lentivirus transduction with viral particles containing pools of tumor suppressor candidate gene gRNAs. Once these pooled, loss-of-function, organoid models were established, they were transplanted into NOD/SCID/γ-chain (NSG) mice for subsequent studies. When taken together, they revealed that loss of function of one or more genes leads to liver metastasis in mice orthotopically implanted with these organoids [33]. Importantly, the system they developed for colorectal cancer is an innovative strategy for manipulating driver genes using organoids that can be leveraged for discovery of other critical driver genes and assessment of therapeutics.

Organoids have also been established from multiple single cells isolated from both normal intestinal crypts and colorectal cancers [34]. In this study, pieces of tumors were isolated from distinct locations of colorectal tumors from three previously untreated patients, grown as organoids, and sorted via flow cytometry to yield single cells for subsequent organoid culture. Phylogenetic trees were constructed using mutational data derived from whole genome sequencing or targeted gene panel sequencing, comprising 360 known cancer genes. Methylation patterns, epigenetic analysis, and drug sensitivity were also examined for the single cell–derived organoids. Interestingly, single cell–derived organoids exhibited extensive genetic heterogeneity. Key putative driver mutations identified as being in the trunk of the phylogenetic tree of one patient included PIK3A (E81K) and BRAF (V600E) together with microsatellite instability and hypermethylation of the MLH1 gene. The second patient had two protein truncating mutations in APC together with a mutated TP53 containing a homozygous splice site mutation [34]. The third patient, on the other hand, had a mutated KRAS (A146T) and two truncating APC mutations. Based on the mutational load and the somatic mutations observed, mutational signatures were identified and applied to each segment of the phylogenetic tree for each of the single cell–derived organoids [34]. Thus, these findings provide additional support for the concept that as cancers develop and evolve, they acquire more somatic mutations compared to normal cells. The processes that permit successive mutation in these colorectal cancer cells likely become more permissive over time; however, the timing remains unclear. Interestingly, as noted above, two of the patient-derived organoids were mismatch-repair proficient [34].

The aforementioned study demonstrates that organoid based systems can be used to construct complex phylogenetic and mutational signature profiles of single cells that appear to recapitulate the mutational dynamics of the tumor. Thus, organoids may be used map mutational processes that could predict tumor responsiveness to the environment or therapeutics over time.

### Platform for drug screening and drug discovery

In addition to their utility in dissecting tumorigenesis and cancer evolution, organoid models can be used to study a tumors response to cancer chemotherapeutic agents.

This generally entails constructing a library or biobank of organoids established from numerous tumor samples or biopsies and performing gene expression profiling prior to drug screening, which is technically complex. The goal is to establish a protocol for creating a patient-derived organoid (PDO) tumor model that faithfully reproduces the genotype, phenotype, and therapeutic response of the patient’s tumor such that it can be used to study new and existing agents, as well as drug resistance.

The combination of gene expression analysis and therapeutic profiling is now more readily being employed to characterize and validate organoids in order to match their biological progression response to treatment with that of the tumor [35]. Studies differ in several important terms that need to be considered: the size of the biobank or library, the outgrowth efficiency from the primary tumor sample, culture conditions, methods used to characterize organoid gene expression, similarity to the primary tumor, the number and types of agents screened, and the therapeutic outcomes.

In general, the size of biobanks or libraries is limited by the availability of tumor samples. Additionally, the method of tumor tissue procurement (i.e., biopsy, surgical resection) can alter the size of the biobank or library due to differences in efficiency of organoid outgrowth and isolation techniques [36]. For example, a recent study constructed a biobank from 83 tumor samples isolated via a combination of surgical resection and biopsy [37]. The overall outgrowth efficiency of the organoids was 62% [38]. The majority of the organoids sequenced were obtained via surgical resection, whereas the outgrowth efficiency of biopsy sourced organoids was low (31%) [37].

Studies on refining the method of establishing organoids from biopsy samples have led to improved outgrowth efficiency [36]. Starting with 159 pancreatic tumor samples from 138 patients, Tiriac et al. ultimately constructed a library of 114 PDOs from 101 of these patients [28]. Slight variations in efficiency depending on the route by which the tumor sample was obtained (fine needle biopsy or tumor resection, 72% versus 78%, respectively) were observed. In addition to the patient-derived tumor samples, the authors developed 11 human normal pancreatic ductal organoids from pancreatic islet transplant samples [35].

Biobanks of organoids can also be used to perform co-clinical trials (a side by side study matching patient drug responses to the drug responses of pre-clinical, translational laboratory models) [37]. In this study, metastatic colorectal cancer (*n* = 16), gastroesophageal cancer (*n* = 4), and cholangiocarcinoma (*n* = 1) were procured via biopsy and grown as organoids, with a 70% efficiency [32]. This biobank then served as a platform for drug screening, discussed more below. In a similar study, Ooft et al. examined the predictive potential of organoids derived from metastatic colorectal cancer biopsies (*n* = 67) from patients (*n* = 61) prior to receiving chemotherapy and compared the patient’s response to drug with that of the organoid’s response to drug [39]. Due to a variety of circumstances (i.e., retrieval of tissue and cells, quality control, and bacterial infection), the authors report that they achieved a 63% PDO culture rate for tumors isolated from patients via biopsy.

In concert with the route of procurement, culture conditions can also have a profound influence on outgrowth efficiency. Serum-free culture conditions can be used for organoid propagation to ensure that nonepithelial cells do not survive culture and propagation efficiency is maximized [35, 40, 41]. Others have looked at how different types of organoid media can be formulated to select for organoids harboring certain oncogenic mutations while eliminating overgrowth of non-tumor cells [37]. For example, conditioned organoid media created selective pressure for outgrowth of KRAS G12R mutant tumor cells from normal pancreatic tissue from an unidentified pre-cancerous lesion in a patient sample [37]. Others have reported growing their PDOs on Matrigel using standard PDO culture media to select LGR5^+^ stem cells from their biopsies with a high rate of efficiency (70%) in line with previously published data [42]. Histological and immunohistochemical assessment is often used to confirm that the organoids retain parental tumor characteristics [32].

Studies are increasingly using combinations of high-throughput methods to document the landscape of gene expression and genetic mutations in organoids as a prelude to interrogating them with drugs. For example, single nucleotide variants (SNVs) and copy number alterations (CNAs), characterized using Sanger sequencing and whole exome sequencing (WES), have been used to establish the genetic and mutational landscape of the organoids PDAC organoids [28]. In this study, whole genome sequencing (WGS) was employed with a subset of the PDAC-confirmed PDOs to determine the degree of similarity between the organoids and matched tumors [28]. Similarly, transcriptomic profiling using RNA sequencing (RNA-seq) has been used to compare the gene expression of the PDOs with classic and basal signatures identified from virtually microdissected PDAC [35, 43]. WGS and next-generation sequencing (NGS) are also used to look at panels of oncogene or tumor suppressors commonly found in a tumor type to validate the organoid model and to guide selection of therapeutics for experimentation [32, 37].

Ultimately, the end goal of these efforts to characterize organoids is to understand the parameters governing sensitivity or resistance to conventionally used drugs and to discover new agents. Indeed, transcriptomic gene expression profiling is facilitating screening of difficult to treat cancers such as PDAC [28]. Pharmacotyping PDAC PDOs using commonly employed conventional chemotherapeutic agents such as gemcitabine, nab-paclitaxel, irinotecan, 5-fluoruracil, and oxaliplatin revealed interpatient variability to these agents [28]. This, in turn, allowed further classification of the PDOs according to degree of chemosensitivity (i.e., most responsive, intermediately responsive, and least responsive). Comparison of these results with retrospective treatment data for patients, from which these PDOs were derived, revealed that the treatment responses as assessed by progression-free survival were similar [35]. Subsequently, the effectiveness of a range of targeted agents was assessed, with several demonstrating efficacy towards chemoresistant PDOs, including selumetinib (MEK 1/2 inhibitor), afatinib (EGFR tyrosine kinase inhibitor), everolimus (mTOR inhibitor), and LY2874455 (FGFR inhibitor) [35]. Thus, incorporating transcriptomic data with drug sensitivity pharmacotyping data provides further stratification of the PDOs and may enable development of novel therapeutic strategies.

Biobanks of organoids can also be used to screen large sets of conventional and targeted agents in tandem with gene expression profiling. Following gene expression profiling of hundreds of genes known to be involved in PDAC oncogenesis, the viability of a bank of PDAC derived organoids was assessed following treatment with a range of agents [37]. In this case, twenty-four PDOs in this bank were used to screen a panel of 76 therapeutic agents revealing a wide range of individual responses to targeted therapeutics and, in the case of a small subset of 4 patients, the PDO responses correlated with clinical responses [37]. Importantly, they queried their PDO system with a novel therapeutic, protein arginine methyltransferase 5 (PRMT5) inhibitor EZP01556 that exploits a synthetic lethal vulnerability of the 80–90% of PDACs that are deficient for the gene MTAP (methyladenosine phosphorylase) [44]. Indeed, MTAP organoid lines exhibited greater sensitivity to PRMT5 inhibitors, although some subsets of MTAP^+^ organoids also responded [37]. This both reinforces the potential of organoid systems for testing novel agents and underscores the need for further study.

Finally, screens of drugs can be done in organoids using treatment protocols that mimic phased clinical trials or use a co-clinical trial design to explore combination therapy [32, 35]. Using a library of 55 drugs in phase I–III clinical trials or currently in clinical use, Vlachogiannis et al. demonstrated the ability of their PDO system to recapitulate drug responses by correlating drug responses of the PDO to that of the patient and tumor genotype [37]. While they found that the PDOs with amplifications in some genes responded to agents targeting the products of those genes, they did note that not all mutations profiled were predictive of response. For example, PI3K mutations did not predict response to the dual PI3K/mTOR inhibitor GDC-0980 [37]. As part of the co-clinical trial, they established orthotopic xenograft mice using organoids and assessed the response to regorafenib, a multi-angiogenic kinase inhibitor [37, 45]. Using MRI imaging in tandem with CD31 immunostaining, they observed a similar response in patients (resistance vs prolonged disease stability) compared with PDOs derived from these patients. They report that their PDO system exhibited 100% sensitivity, 93% specificity, a positive predictive value of 88%, and negative predictive value of 100% to targeted agents or chemotherapy, suggesting the potential utility of such pre-clinical systems for drug screening and activity prediction [37].

In another recent example, CRC PDOs were randomized into treatment arms: 16 PDOs to standard first-line therapy with 5-FU and oxaliplatin; 12 PDOs to second-line therapy with 5-FU and irinotecan; 10 PDOs to single-agent irinotecan. Following this, they created a classification model to predict non-responders to monotherapy with irinotecan. Analysis of growth rate inhibition metrics and dose response curves for PDOs (representing both progressive disease and stable disease) after treatment with irinotecan provided them with a training data set they could query using a model prediction method called leave-one-out-cross validation (LOOCV) [39]. This allowed them to classify 80% of non-responders from organoid drug sensitivity data compared with the corresponding patient source. They note that this assay only required 5000 cells to perform. Similar classification of combination therapy with 5-FU and irinotecan also suggested that PDOs have predictive value for combination therapy. In this case, to generate the complementary dose response curves for the 5-FU and irinotecan analysis only required 10,000 cells and could be generated in 21 days, a vast improvement over traditional cell culture systems [39]. Interestingly, they showed that their organoid system failed to predict response to the combination of 5-FU and oxaliplatin. This is an important result because, as they note, it reveals the limitations in modeling combination therapy responses in organoids [35]. This may be a result of incomplete understanding into the nature of the synergism between agents or could also be due to a lack of certain biological components in the organoid system such as metabolizing enzymes (i.e., cytochrome P50s) or the microbiome.

As discussed, many studies have focused on validating the organoid models, building biobanks, or libraries, and screening them with a small cadre of predominately known therapeutic agents [28]. However, what remains to be determined is whether organoid-based systems can actually facilitate the discovery of novel (and active) therapeutic agents. Creation of novel systems or strategies to propagate and curate organoids for drug screening may provide an avenue to successful identification of novel cancer drugs. Indeed, a novel miniaturized organoid culture method that incorporates mini-rings for 3D culture that can be rapidly screened for drug sensitivity (within 1 week from surgical resection) was recently described [46]. This system uses fewer cells, smaller amounts of Matrigel, and entails seeding cells around the rim of a 96-well plate in a ring shape using a single well or multi-channel pipette. Additional advantages of this system are that adding and removing media and drugs can be done with minimal disruption to the cells and the organoids appear to resemble the tumor from which they were derived. Small-scale proof-of-concept experiments using doxorubicin, staurosporine, and a novel peptide inhibitor of p53, ReACp53, revealed that the Matrigel layer allowed both small molecules and peptides to penetrate and reach the organoids. A larger screen with 240 protein kinases, at two different concentrations, was performed using organoids derived from 4 patients with ovarian and peritoneal tumors. These included inhibitors of CDK, MEK, EGFR, PI3K/mTOR, IKK, HDAC, and Flt [46]. In general, they reported tumor-specific, non-redundant responses for the inhibitors they assayed with the exception of BGT226 (an PI3K/mTOR inhibitor) which elicited responses from all of the organoids screened. Thus, in accordance with the other studies discussed so far, organoids appear to be a reproducible platform for personalized drug screening that appropriately recapitulates the challenges of inter-and intra-tumor heterogeneity observed in patients. This system, which does appear cheaper and faster, could potentially be deployed at a scale which could yield more statistically robust and clinically translatable results.

The recently reported use of single-cell techniques to establish organoid cultures is another promising approach to determine if the intra-tumor heterogeneity seen in individual cancer cells translates into differential treatment responsiveness [34]. This is supported by the observation that organoids can stably retain the genetic and epigenetic during propagation [29]. As a consequence, derivation of organoids from single-cells can shed light on how drug resistance develops in tumors, which may occur late in tumorigenesis at geographically distinct sites in the tumor [27].

The advent of organoids-on-a-chip, which seek to replicate *in vivo* conditions *in vitro*, using organoids is particularly exciting [47]. This technology offers the opportunity to assess cancer cell interaction with normal tissue, the immune system, and response to therapeutics. A model to study the interactions of T cells with tumor organoids established from mismatch-repair deficient CRC or non-small-cell lung cancer (NSCLC) was recently reported [48]. In this system, co-culture of peripheral blood lymphocytes with tumor organoids led to enhanced T cell tumor reactivity and cell killing [48]. Furthermore, T cell reactivity was exclusive to the organoids that had been previously co-cultured, indicating and that this system may be utilized to assess the extent of immune-specific cytolytic T cell–mediated tumor destruction [48]. Other studies also lend support to the use of organoids in elucidating immune-tumor interactions further suggesting that organoid models could be adapted to an organoid-on-a-chip format together with stromal cells and microvasculature [42].

Another application of organoids-on-a-chip technology could be in assessing drug-induced target and non-target organ toxicity, a frequent cause for clinical drug failure [47, 49]. For example, Skardal et al. created a triple-tissue organ-on-a-chip platform in which they integrated heart, liver, and lung organoid systems through perfusion-driven microreactors constructed on poly-dimethyl siloxane that was connected serially under fluid flow [47, 49]. In the “liver-on-a-chip” component of this integrated system, they show stable and reproducible activity of stellate cells, Kupffer cells, and hepatocytes with measurable ATP production. Remarkably, this system can also be used to measure output of products such as albumin and urea, harbors metabolically active cytochrome P450 enzymes (CYP3A4 and CYP2C19), and it can be used to assess liver damage following exposure to acetaminophen with lactate dehydrogenase levels and liver cell protein release as outputs of drug-induced related liver toxicity [47, 49]. This last point is crucial, as this team demonstrated superior advantages of this system compared with 2D hepatocyte culture, and highlights the clinical relevance of this novel approach in predicting liver metabolism and toxicity which are critical components of the drug development process [47, 49].

Finally, an area of need that could be addressed with organoids-on-a-chip is modeling the effect that biochemical and metabolic intermediates have on tumors and their extracellular environment. In other words, can the non-cellular conditions in which a tumor resides and proliferates be recapitulated? Some promising work along these lines has appeared in the literature in regard to tumor microenvironment metabolites but more progress is needed [50, 51]. Advancements in microfluidics and perfusable blood vessels permit incorporation of soluble factors and nutrients, normally present in the extracellular milieu, into these models that more accurately mimic *in vivo* culture conditions and tumor microenvironments [47, 52].

In summary, organoids, like the human patients they are derived from, exhibit genotypic and phenotypic heterogeneity, in some cases, organoids recapitulate clinical responses. Establishment of organoid biobanks and libraries allows high-throughput compound screening that can be readily translated into animal models.

## Mouse models

Tumor development is, in general, a progressive process fueled by mutations in driver genes such as oncogenes or tumor suppressor genes that ultimately provide an evolutionary advantage for tumor survival [53, 54]. Considering the number of driver genes that can be mutated, it is not surprising that tumors are heterogeneous, containing many sub-clones that take different paths during clonal evolution in a self-selecting, self-propagating manner. In addition to genetic changes, the tumor microenvironment, the immune system, exogenous toxins and environmental xenobiotics, cancer chemotherapy, and the microbiota can all influence the mutational course a tumor traverses as it grows and evolves. Therefore, having a model system whereby biological consequences of step-wise mutational changes can be mapped, and future mutations predicted, is of great importance. Gaining insight into this process could aid in drug discovery and tailored treatment of patients. In the sections that follow, we will review the different mouse models currently used in cancer research and discuss representative studies, advantages, and disadvantages of each system. For each of the models, we will consider its suitability for studying: tumor evolution and heterogeneity, metastasis, immune-tumor interactions, and suitability as a platform for drug discovery and screening (Fig. 1).

### Xenograft and syngeneic mouse models

For decades, the most basic and frequently employed mouse models used to assess tumor growth and screen conventional chemotherapy or candidate drugs have been simple xenografted or syngeneic mice. Typically, these mouse models involve subcutaneous administration of human (xenograft) and mouse (syngeneic) tumor cells without regard to the organ of tumor origin (heterotopic) or via implantation of tumor tissue or cells into the tissue corresponding to the site of the tumors origin (orthotopic) [55]. Cell culture–derived xenograft mice and syngeneic mice require less technical skill and time to establish but they are less predictive of a patient’s response to therapeutics compared with genetically engineered mouse models (GEMMs) or patient-derived xenograft (PDX) mice, which are discussed in subsequent sections [56].

#### Tumor evolution and heterogeneity

For a variety of reasons, simple subcutaneous xenograft mouse models, where human tumor cells grown on a plate are injected into a mouse, are not robust tools in investigating tumor evolution [55, 57]. This is, in part, due to the source material. As discussed, cancer cells grown on plates are unreliable predictors of mutational progression in tumors due to numerous additional mutations cell lines acquire from repeated propagation on artificial tissue culture plates [12, 58]. Indeed, cancer cell lines tend to lose their heterogeneity and become more homogeneous with continued selection on cell culture media in the absence of tumor microenvironment and immune influences [57, 59]. Orthotopically implanting human or mouse cancer cells into a mouse yields a more representative model of tumor development because the implanted tumor is placed in the organ environment similar to that from which it originally arose [55, 57]. In the case of some tumor types, such as breast cancer, the orthotopically implanted tumor fragments are more representative models of tumor development and tumor microenvironment [55, 57].

Syngeneic mouse models represent an improvement on heterotopic subcutaneous or orthotopic xenograft models using human cancer cells to study tumor evolution and tumor heterogeneity because murine tumor cells can be inoculated or implanted into immunocompetent mice [60]. Syngeneic mice were among the first *in vivo* oncologic models created, with the discovery of the mouse leukemia model L1210 in 1960 followed closely by establishment of solid tumor syngeneic models [58]. This leukemia model, studied extensively by Howard Skipper for drug and pharmacokinetic screening, demonstrated not only that combination chemotherapy could be superior to single treatment experiments, but also that the schedule of administration was important to the efficacy of a given regimen. In 1964, Skipper would report the cure of murine leukemia in L1210, a discovery that aided in the development of a durable cure for acute lymphocytic leukemia in a subset of human patients [61]. Although many years have passed since this milestone, syngeneic mice continue to be used as a pre-clinical models for drug screening, and more recently as essential tools for predicting response to immunotherapy [58, 61–63]. With their wide application potential, it is important to consider specific molecular features when selecting a model for drug screening, pharmacokinetic studies, and translational potential.

Unfortunately, syngeneic mouse tumors have the disadvantage of a lower mutational load than comparable human tumors [57]. In general, mouse tumors are less heterogeneous than human tumors because of inter-species differences [9, 64]. This ultimately translates into differences in gene expression profiles, baseline immune infiltrates, and response to drug treatment including immune checkpoint blockade [58, 61]. Indeed, analyses of syngeneic tumors of colon, breast, renal, and melanoma origin have revealed that there are profound differences between models even of the same cancer type, indicating that extensive profiling of syngeneic models may be necessary for creation of more predictive pre-clinical models [61]. In a study of 12 different syngeneic orthotopic models of metastatic breast cancer, differences in gene expression profiles, histopathology, angiogenesis, and proliferation rates have also been noted [65]. Despite this inter-model heterogeneity, these syngeneic mice recapitulated human samples and more than half of the most commonly mutated genes in human breast cancer were represented within their panel and could assist in predictive modeling for different forms of breast cancer [65].

#### Metastasis

The suitability of mouse models for studying metastasis continues to be a challenging area. Unsurprisingly, heterotopic mouse models where cancer cell lines are injected into a mouse rarely result in metastasis [65–67]. This is, in part, a product of poor mismatch between the cancer cells and the tumor microenvironment of the mouse and the lack of an immune system (i.e., nude mice and NSG mice) [55, 66, 67]. This is especially true for xenograft models, where human cancer cells are injected into a mouse; however, similarly poor rates of spontaneous metastasis are encountered with allograft injection of mouse cancer cells into mice [66, 67]. Interestingly, in the case of intravenous injection, the site of injection dictates to some degree the extent and site of metastasis [66]. Injection of cancers cells in the tail vein of the mouse results in metastasis to the lung, portal vein injection leads to hepatic metastasis, intracardiac injection yields more diffuse metastasis to brain and bone, and so on [66, 67]. Thus, it stands to be questioned if this truly represents metastasis, as the early steps in metastatic development and progression are bypassed due to the route of administration [66, 67]. There are, however, a handful of examples where injection of human cancer cells leads to metastasis. These include metastatic prone human cancer cells lines such as MCF-7 and MDA-MB-231 breast cancer cells, KM12 colon cancer cells, A7 and B16 melanoma cells, PC-3 prostate cancer cells, and SKOV3 ovarian cancer cells [66].

In contrast to heterotopic administration of cancer cells, orthotopically implanting human cancer cells or mouse tumors cells into mice more closely approximates the tumor microenvironment and can, in certain syngeneic mouse models, result in metastasis [65]. There are a number of mouse cell lines that lead to tumor formation and metastasis such as 4T1, B16, Lewis lung carcinoma cells, Met-1, and RM1 [66]. It was recently reported using orthotopic injection of 4T1 mouse mammary tumor cells (generated from CARMIL1-WT or CARMIL1-AA cells) into BALB/c mice to study the role of macropinocytosis in mediating treatment resistance [68]. They found that macropinocytosis fuels tumor growth (possibly by generating intracellular cell nutrients such as amino acids, sugars, fatty acids, and nucleotides via necrocytosis) and increases resistance to 5-FU [68]. Despite these examples, it is still not clear whether these models are more predictive when utilized to assess anti-cancer therapy.

#### Immune-tumor interactions

Of all of the models discussed, xenografts are the least useful for studying immune-tumor interactions [66]. Syngeneic mouse models do have an intact immune system, although it is a mouse immune system and may not approximate the types of interactions observed in human tumor environments (particularly in regards to evaluating biologics targeting human proteins such as PD1) [69]. Nevertheless, there are advantages to using parenterally or orthotopically derived syngeneic mice including short latency periods, reproducibility, and genetic tractability [55, 70]. There are many examples of syngeneic mouse models that have been used to explore immune-tumor interactions [71–74]. To address potential differences between these models for immunotherapy, Mosley et al. meticulously compared syngeneic mouse models through the lens of immunologically hot and immunologically cold tumors that involved gene expression profiling of immune-related pathways and responses to immune checkpoint inhibitors [71]. The hot or cold phenotype correlates with the extent of T cell infiltration into to the tumor microenvironment [75, 76]. They began by assessing the effectiveness of inhibiting immune checkpoints with anti-CTLA-4 antibodies or anti-PD-L1 antibodies in six different syngeneic mouse tumor models (CT26, RENCA, 4T1, B16F10 AP-3, LL/2, and MC38) and observed that only two models responded to the anti-CTLA-4 treatment, CT26 (a mouse colon carcinoma model), and RENCA (a mouse renal cell carcinoma model), as measured by decreases in tumor volume [64]. Only the CT26 mice responded to anti-PD-L1 treatment. Based on this differential response, the authors performed genomic analysis of the original cell lines used for these mouse models by examining CNVs, whole-exosome analysis, and transcriptomics to look for differences in gene expression signatures and mutational profile between the different models. They discovered that CNV levels in the parent cell lines were not altered by their method of generation and that, in the case of CT26, there was no difference in the overall mutational burden [71]. Using a panel of 64 prominent cancer genes, the authors then used WES to compare the mutational profile of tumor cell lines with matched tumors. In the case of CT26, they found that *APC* and *Kras* were mutated in both CT26 cell lines and human colorectal tumors but that CT26 did not have the *Trp53* mutation found in human colorectal tumors. Nevertheless, they found a high overall correlation (*r* = 0.766) in mutant allele frequency between the murine cell lines and the corresponding syngeneic murine tumors (*in vitro* versus *in vivo*) [71]. Transcriptomic profiling revealed that differences in gene expression in innate immune-related pathways could have accounted for the lack of responsiveness to checkpoint blockade and the cold phenotype of B16 mouse tumors. Further support for differences in the immune system microenvironment between these models came from analysis of immune cell infiltration in tumors via flow cytometry where they profiled nine nonoverlapping innate and adaptive immune cell phenotypes [71]. This analysis demonstrated that immune cell infiltration varied markedly between the models perhaps accounting for the differential responses. For example, 4T1, MC38, and LL/2 were enriched for immunosuppressive granulocyte macrophage–derived suppressor cells (gMDSCs) and monocytic macrophage-derived suppressor cells (mMDSCs), whereas B16F10 and AP-3 tumors were poorly infiltrated by immune cells overall and therefore immunologically cold. Importantly, CT26 and RENCA had the most balanced population of immune cells including the highest CD4^+^ and CD8^+^ T cell populations of all the models indicating a robust immune reaction even in the absence of immunotherapy [71]. Lastly, they note that while these models may not clearly translate to the clinic, they do provide a platform to evaluate the ability of immunotherapeutic approaches to achieve responses in tumors with a different immunophenotypic background.

Finally, due to the abundance of well-established models and the advantage of an immunocompetent system, syngeneic models have been widely utilized as a pre-clinical tool for immunotherapy screening and it is thus important to characterize whether pharmacokinetic (PK) and pharmacodynamic (PD) parameters can be accurately translated into human clinical trials. Using the syngeneic MC38 tumor–bearing C57BL/6 mice, it was demonstrated that this model was useful for optimizing dose-range selection for the anti-PDL1 antibody pembrolizumab in early clinical development. As a result, they concluded that antibody distribution kinetics, drug association and dissociation, receptor occupancy, and dose response to a wide range of tumor growth rates were all measures that could be allometrically scaled to human parameters and accurately simulate findings in the clinical setting for selection of the lowest effective dose [77].

#### Drug discovery and drug screening

Xenograft and syngeneic mouse models have been extensively used as a means to assess the ability of conventional therapeutic agents to alter tumor growth or volume [56, 60, 78–86]. However, there appears to be divergence among xenograft and syngeneic mouse models in terms of their responsiveness to conventional chemotherapeutic agents. A retrospective based literature search comparing the responses of cell lines, xenograft, and syngeneic mice to thirty-one different cytotoxic cancer drugs found markedly distinct outcomes that differed across tumor types. For the four solid tumor types examined, colon, breast, ovarian, and NSCLC, they correlated the pre-clinical *in vitro* activity of each drug with phase II response rates by tumor type [56]. They also calculated whether the response in one tumor type could predict response in the same tumor type, in the other three tumor types combined, or in all four tumor types combined. In this case, it was found that cell lines could predict response in NSCLC, breast, and ovarian cancer, whereas syngeneic mouse models were not predictive and cell line–derived xenografts were predictive for NSCLC and ovarian cancer, but not breast cancer and colon cancer. In contrast, a screen of seven different syngeneic models of varying cancer types (two leukemia models and five solid tumors) found that syngeneic *in vivo* models can be used as a platform for drug screening, particularly for lymphoma, melanoma, and breast cancer [62].

Much like drug response, the predictive value of syngeneic models for assessing drug pharmacokinetic and pharmacodynamic parameters is dependent on the syngeneic model and tumor type being studied. In lymphoma syngeneic models, for example, the pharmacokinetic properties of rituximab plasma concentration and overall efficacy are significantly influenced by tumor burden in mice similar to what is encountered in human clinical studies [87].

However, when taken together, the predictive utility of this cell-derived xenograft and syngeneic mouse models for drug response differs significantly between cancer types, with some positive results in melanoma and lymphoma, and disappointing results in other cancer types such as colon, breast, ovarian, and lung cancer [62, 63]. Further, even within the same cancer type, different syngeneic tumor cell lines have demonstrated markedly different molecular features and immunologic profiles in breast cancer, as well as renal and colon cancer [71, 88]. An additional concern regarding this model system is the lack of an accurate tumor microenvironment with stromal, vascular, and immune components when using either flank or orthotopic injection [89]. Insofar as these specific cancer types are concerned, thorough interrogation of a particular syngeneic model regarding gene expression pattern and immunologic profile within the context of the human target population is required.

To further enhance the mechanistic and clinical relevance of pre-clinical *in vivo* models, GEMMs and PDXs models were developed.

### Genetically engineered mouse models

Genetically engineered mice, or transgenic mice, were first described in the early 1980s following development of techniques that allowed stable transmission of genes to successive generations upon injection of cDNA into mice pronuclei [90]. This technique led to establishment of models of oncogenesis whereby oncogenes were overexpressed, or tumor suppressor genes silenced and this, in turn, yielded spontaneous tumor formation. The earliest studies with genetically engineered mouse models (GEMMs) demonstrated that inserting oncogenes such as ERG, KRAS and MYC, for example, into transgenic mice led to the development of cancer [91, 92]. Thus, tumor development in these models was driven by genetic manipulation. As will be discussed, newer methods including Cre/loxP gene silencing, viral vectors, or CRISPR/Cas9 gene editing have emerged that dramatically alter the time scale of producing GEMMs [70]. GEMMs can also be induced to develop spontaneous tumors upon exposure to environmental factors (i.e., carcinogens, radiation) which can induce single nucleotide changes in genes and recapitulate a patient’s tumor [57, 93, 94].

Some of the notable limitations of GEMMs are that generating and propagating them requires dedicated labor, is time consuming, expensive, and as already discussed there is less resemblance to human tumors. However, GEMMs can play an important role in dissecting out specific molecular events in oncogenesis and in determining the relationship between these events and therapeutic responsiveness.

#### Tumor evolution and heterogeneity

GEMMs are, arguably, a very effective mouse model for studying tumor heterogeneity and evolution, because they are genetically tractable, allowing investigation of specific mutations in a stable genetic background [57]. As noted, GEMMs are classically generated by inserting inducible or constitutively expressed cDNA, encoding tumor suppressor genes or oncogenes, into mice via direct injection of mouse oocytes or by means of viral vectors [57, 95]. More recently, targeted disruption of genes using Cre/loxP recombinase has been developed that allows conditional expression or deletion of genes.

Niknarfs et al. used transgenic GEMMs to characterize clonal evolution in pancreatic cancer [96]. The two GEMMs, known as KPC (LSL-Kras^G12D/+^; LSL-Trp53^R172H/+^; Pdx1-Cre) and KPTC (LSL-KRASG12D/^+^; LSL-Trp53R172H/^+^; Tgfbr2flox/^+^; Ptf1aCre/^+^) are conditional mice that when bred with cre-recombinase-expressing mice, lead to expression of active mutant Kras, and loss of p53 (by means of a dominant negative mutation) [96]. These mice closely mimic the histopathology and clinical features of PDAC and are more biologically relevant models for studying clonal evolution and tumor heterogeneity [96]. Based on their results, these authors concluded that KPC and KPTC mice accumulate sub-clonal somatic mutations as measured by copy number alterations in essential PDAC pathways including chromosomes containing the Cdkn2a gene (chr4), Tgfbr2 gene (chr9), and Trp53 (chr11) [96]. Other modifications in DNA damage response genes (Msh3), and genes involved in cellular recovery to DNA damage (Mastl) were also noted [96]. This group also discovered an unrecognized role for the gene Nlrp1b (part of a family of immune pattern recognition receptors) that was found to have undergone focal homozygous deletion in three mice (KPC8, KPC9, and KPTC26) [96–98]. Nlrp1b is also somatically altered in human pancreatic cancers. Ultimately, by modeling the genomic mutations observed in their models across different segments of the tumors, they could also assess the spatial heterogeneity within each of the mouse tumors allowing them to construct phylogenetic trees charting the evolutionary history of cells from each segment [96]. However, a liability of this method is that it can alter the germline. With more recent CRISPR/Cas9 techniques, researchers can move away from blunt germline alterations of gene expression to more controlled, targeted tissue-specific changes. Indeed, the use of CRISPR/Cas9 gene editing is now enabling manipulation of numerous cancer genes simultaneously [99].

As was discussed with organoids, single-cell analysis is an emerging tool that can be used in tandem with mouse models such as GEMMs to trace cell lineages with applications in cancer [100, 101]. Also, the applications of these combinations of technologies can be used to address tumor heterogeneity in non-cancer tumor cells, such as stromal cells. For example, Bartoschek et al. used single-cell analysis to identify spatially and functionally distinct classes of cancer-associated fibroblasts from a MMTV-PyMT mouse breast cancer model [102].

#### Metastasis

Compared with other mouse models, GEMMs are well suited to investigating metastasis and there is a growing body of literature demonstrating *de novo* metastasis of breast and pancreatic cancer tumors [66, 103, 104]. This is highlighted in studies using the KPC pancreatic adenocarcinoma transgenic mouse model, which carries both inactivating p53 and activating KRas mutations [55, 105]. As a result, these mice develop spontaneous tumors which metastasize to the liver at a high rate of 50–75% of mice [55]. In addition, in contrast to patient-derived xenograft or syngeneic mouse models, these mice also exhibit significant stromal and fibroblast infiltration, similar to that observed in human pancreatic adenocarcinomas [105]. These features make the KPC GEMMs useful models for studying the biology of metastasis, anti-metastatic agents, and treatment resistance [55, 105, 106]. For example, Morton et al. showed that the Src inhibitor could inhibit metastasis in KPC GEMMs [92]. There are a number of other GEMM models of PDAC with mutant p53 and Kras which also have fibrous desmoplastic stroma (that interferes with both drug penetration and immune infiltration), as well as PDAC GEMM models with PanIN cells that undergo epithelial-mesenchymal transition (EMT), further supporting their clinical relevance [9, 107–109].

Finally, a number of recent studies have used GEMMs to delineate mechanisms of metastasis in small-cell lung cancer (SCLC) [110–112]. Building on the observations that p53 and RB are mutated in more than 90% of SCLCs, mouse models were established where these genes could be conditionally silenced using the Cre/loxP recombination [113]. Deletion of the cell cycle inhibitor, p130, in this model leads to enhanced tumor development [113]. Denny et al. used loxP-flanked Trp53^f/f^, Rb1^f/f^, p130^f/f^ mice crossed with R26^mT/mG^ mice to establish conditional knock-down transgenic mice that, when administered adenovirus containing the cre-recombinase via inhalation, were deficient for p53, Rb1, and p130 [110, 114]. These mice, in turn, develop tumors that are tomato (mT) negative and GFP positive owing to the cre-mediated excision of mT in p53/Rb1/p130-deficient tumor cells, but not in normal cells [110, 114]. In this way, it was possible to track and visualize GFP-positive tumor cells that metastasized. Using this system, the authors were able to identify a putative driver of metastasis, the transcription factor Nfib, which is involved in chromatin remodeling [110]. This same study also employed NSG mice to verify the metastatic potential of high and low Nfib levels via subcutaneous administration or transplantation of SCLC cells deficient in or overexpressing Nfib [110]. Other groups discovered a similar relationship between Nfib and SCLC metastasis using Trp53, Rb1 conditional knock-down GEMM mouse models [111, 112]. These are just a few specific examples demonstrating that GEMMs may be a useful for studying metastasis.

GEMMs may also be utilized to study epigenetic regulation of metastasis. Recent studies show that epigenetic modification of immune cells can serve as a precondition for premetastatic microenvironment establishment and metastasis initiation [115]. These studies highlight how different mouse models can work in tandem to overcome limitations of any one model. For example, a combination of GEMMs, xenograft mice, and NSG mice were used to assess the effectiveness of epigenetic modifying drugs on myeloid-derived suppressor cells (MDSCs) in mediating metastasis [116]. First, syngeneic C57/BL/6 mice or BALB/c mice were either subcutaneously or orthotopically implanted with different cell lines known to aggressively metastasize to the lungs: Lewis lung carcinoma cells (LLC) (Sub-Q into C57/BL/6 mice), HNM007 esophageal squamous cell carcinoma cells (Sub-Q into C57/BL/6 mice), or 4T1 mammary cancer cells (orthotopic injection into mammary fat pads of BALB/c mice) [116]. Tumors were resected and metastasis was assessed by immunofluorescence and histology. Next, CD45.1 MDSCs were isolated from the LLC or HNM007 tumor–bearing mice and transplanted into mice congenic at the CD45 Ly5 locus (B.6SJL-*Ptprc*^*a*^ *Pepc*^*b*^/BoyJ Ly5.1) [116]. The NSG mice transplanted with LLC tumor tissue were used as a model to guide dosing with the epigenetic modifying drugs azacitidine and entinostat. Host MDSCs derived from CD45.1 mice-bearing LLC tumors were then adoptively transferred into CD45.2 congenically marked mice via tail vein injection in order to track the primed tumor–derived MDSCs [116]. Treating these mice with low dose epigenetic modifying drugs led to a reduction in trafficking of MDSCs to premetastatic sites together with enhanced disease-free survival [116]. This was confirmed using B6.129S4-*Ccr2*^*tm1Ifc*^/J mice, a knockout mouse lacking expression of the Ccr-2 gene, an important regulator of monocyte migration from the bone marrow to the tumor microenvironment [116]. Therefore, by leveraging the advantages of each of these mouse models, they demonstrated that epigenetic changes in MDSCs prime them to develop premetastatic niches for tumor cells even after tumor removal.

Another recent example focuses on the role of the epigenetic modifier polycomb repressor complex 2 (PRC1) in mediating stemeness, metastasis initiation, and local tumor immunosuppression in double negative prostate cancer (DNPC) [115]. A combination of mouse models was utilized including nude mice, NSG mice, *Pten*^*PC*^*−/−*FVB/NJ mice and *Pten*^*PC*^*−/−Smad4*^*PC*^*−/−*FVB/NJ mice [115]. The nude mice were used as the model for metastasis due to the lack of an immune system. Nude mice were injected intracardially with luciferase-labeled androgen receptor (AR)–negative PC3M cells in the presence or absence of RNF2 (ring finger 2) shRNA (small hairpin RNA), a catalytic component of PRC1, thereby knocking down PRC1. Silencing RNF2 suppressed metastasis. The *Pten*^*PC*^*−/−* mice develop prostate cancer but have little to no metastasis, whereas the genetically modified *Pten*^*PC*^*−/−Smad4*^*PC*^*−/−* develops metastatic prostate cancer [117, 118]. This provides a model for studying how PRC1/RNF2 alters prostate cancer cells to influence metastasis. Briefly, shRNA-silenced prostate cancer cells from *Pten*^*PC*^*−/−Smad4*^*PC*^*−/−* lacking functional PRC1 were injected into nude mice and metastasis to the bone and liver was reduced. Through a combination of RNA-seq and chromatin immunoprecipitation (CHIP) sequencing, they identified CCL2 as the target of PRC1 that mediates metastasis initiation and immunosuppression. CCL2 promotes recruitment of MDSCs and tumor-associated macrophages (TAMs) that create an immunosuppressed tumor microenvironment and favor bone colonization in prostate cancer [115]. Finally, FVB/NJ mice were inoculated with cells from their prostate cancer model (*Pten*^*PC*^*−/−Smad4*^*PC*^*−/−*) and treated with a small-molecule inhibitor of RNF2, identified in a screen of a compound library–inhibited metastasis. Combination of the RNF2 inhibitor (GW-516) with anti-CTLA-4 and anti-PD-1 antibodies completely suppressed metastasis. Therefore, again, multiple mouse models can be used in tandem to study epigenetics, metastasis, and test novel compounds.

Lastly, an important caveat for congenic GEMM mouse strains that should be mentioned is that they appear to harbor significant amounts of passenger mutations as a consequence of genetic variation from embryonic stem cells (ESCs) used to establish the mouse lines [119]. While this likely poses less of an issue for the vast majority of translational cancer mouse studies, this could have the potential to interfere with interpretation of results in studies using congenic mice particularly as it relates to studies focused on tumor heterogeneity and identification of driver genes.

#### Immune-tumor interactions

Like syngeneic mouse models, GEMMs have an intact immune system (albeit a mouse immune system) so they can more accurately model the interaction of tumors and the immune system in terms of tumor development and response to therapeutics [69]. This is in part due to the native development of the tumors in a microenvironment that adapts and changes with the tumor [89]. This microenvironment contains stromal elements, vasculature, and immune cells that influence the tumor’s relationship with the immune system [89] However, there are a number of limitations of using GEMMs in immune-tumor interaction studies that need to be considered [89]. Due to the fact that these mice are genetically modified, there can be substantial variability in the tumor genotype-phenotype penetrance and latency of tumor development [89]. Also, tumor monitoring and therapeutic response in GEMMs are, in large part, assessed by non-invasive imaging [89]. Consideration also needs to be paid to the consistency of immune targeting between the GEMM murine tumor model and the corresponding human tumor targets, which could affect clinical translation particularly for the development of immunotherapeutic vaccines [89]. However, there is a growing body of literature using GEMMs to study immune-tumor interactions ranging from T cell function, immunogenicity of tumors, and B cell contributions to tumor development and treatment response [70, 120].

Single-cell analysis is also being used along with GEMMs to study immune-tumor interactions. Single-cell analysis has been used to explore the relationship between immune cell infiltration and tumor progression in prostate adenocarcinoma GEMMs deficient for Pten and Smad4 [121]. Loss of PTEN has been shown, in certain cancer models, to be associated with an immunosuppressive tumor microenvironment [89]. In their study, Wang et al. identified MDSCs as the major infiltrating immune cell in tumors from these mice, whose recruitment to tumors is driven by tumor production of the chemokine CXCL5. Single-cell analysis was performed on cells isolated from *Pten*^pc^−/−*Smad4*^pc^−/− mouse blood, lymph, spleen, and primary tumors. The single cells were then immunophenotyped and a tree of cell types isolated from the aforementioned sources was established revealing a striking increase in MDSCs. They went on to identify the underlying signaling pathways mediated by Hippo-Yap1 in the altered expression of CXCL5 in these tumors. Inhibition of the CXCL5 receptor on MDSC or inhibition of YAP1 led to reduced migration of MDSCs to the site of the tumor [121]. Along these lines, there are a number of other recently published studies that have used single-cell techniques in tandem with GEMMS in detailing immune-tumor interactions in breast cancer and lung cancer [102, 122].

#### Drug discovery and drug screening

GEMMs may serve as a useful platform for drug discovery, assessment of drug efficacy, and as a tool to assess multi-organ system adverse effects [55, 70]. GEMMs can also be used to assess how existing and emerging drugs can alter the mutational profile of tumors and lead to treatment resistance [55, 70]. For example, Mitrofanova et al. conducted a study using GEMMs to correlate expression levels of prostate cancer driver mutations such as FOXM1 and CENPF to those in human prostate cancer databases to predict drug response by targeting these specific mutational drivers [123]. They demonstrated that treatment-responsive genes modelled utilizing GEMMs can be used to identify patients that are likely to benefit from treatment with drugs that co-target specific pathways such as the MAP-kinase or mTOR signaling pathways [123]. In addition to targeting a specific signaling pathway, these models can also be used more widely to predict drug response to a number of different inhibitors from various classes. Chesi et al. performed a study in which they used Vk*MYC multiple myeloma transgenic mice to predict responses to six different classes of inhibitors, and demonstrated that drug response was indicative of clinical activity, with a positive predictive value of 67% in associated clinical trials [124]. The study also reported a negative predictive value of 86% for clinical inactivity, indicating that this model may also have translational value for prediction of both drug response and resistance [124].

To overcome some of the limitations of GEMMs, validate mutational changes and results of drug screens, GEMM models can be used in tandem with other mouse model systems such as PDX (discussed below). For example, Liu et al. recently described the development of a triple negative breast cancer (TNBC) GEMM [125]. They generated two cohorts of mice: a *Brca1*-deficient line (K14^cre^, Trp53^flox/flox^, Brac1f^lox/flox^) and a Brca-proficient wild-type line (K14^cre^, Trp53^flox/flox^, Brac1f^wt/wt^) [125]. WES and RNAseq together with copy number alteration analysis of the TNBC tumors revealed several focal amplifications on chromosomes 6 and 9 involving the *Met* and *Yap1* loci which corresponded to elevated mRNA levels for both [125]. They also found that several of the tumors expressed *Fgfr2* and *Raf1* fusion genes, both apparently products of chromosomal translocations. These genetic changes favored oncogenesis via corresponding changes in signal transduction pathways such as MAPK and PI3K, thus revealing therapeutic opportunities [125]. To test the response of these tumors to therapeutic agents, they transplanted the TNBC tumors into nude mice and assessed their response to several targeted drugs. In the case of tumors which spontaneously acquired the *Fgfr2* or *Raf1* fusion proteins, they tested an FGFR inhibitor NVP-BGJ398, the MEK inhibitor trametinib, or the MET inhibitor crizotinib [125]. They demonstrated that tumors with acquired *Fgfr2* fusion proteins were initially responsive to single-agent FGFR inhibition with NVP-BGJ398 but developed resistance in three of six models with an average return to initial tumor volume of 43 days. They further went on to show that the combination of NVP-BGJ398 with Olaparib (a PARP inhibitor) resulted in complete responses with no relapse in all six of the models [125]. This study exemplifies how multiple models (cell culture, GEMM, and orthotopic nude mice) can be used in tandem to explore specific facets of tumor biology and response to treatment.

Beyond utilization as a simple screening platform for pharmacologic response, GEMMs have shown value for modelling drug pharmacokinetic and pharmacodynamic properties, and in some cancer sub-types may be superior to other *in vivo* models. Combest et al. assessed the pharmacokinetic properties of carboplatin in mouse melanoma models and compared results between PDX mice and GEMMs with those observed in the clinical setting [126]. Their findings demonstrate that although the carboplatin plasma PKs of each model were similar, the carboplatin concentration in tumors of the GEMMs more closely resembled those of melanoma patients as compared with the xenograft (A375) model to a significant degree, with a murine-to-human tumor extracellular fluid (ECF) drug concentration ratio of 0.13 and 0.86 for the xenograft or transgenic model, respectively. [126]. This example highlights the need for more studies than model pharmacokinetic drug properties in mouse tumor models.

### Patient-derived xenograft mouse models

Patient-derived xenograft models were first created and published in 1969, when Rygaard and Polvsen first minced, and then injected a colonic adenocarcinoma sample from a 74-year-old patient into athymic nude mice [127]. This model, established for the first time more than five decades ago, has several distinct features when compared with cell line–derived xenografts and syngeneic or transgenic mouse models. Established as a useful tool for translational research, PDX models have the advantage of maintaining the cellular and histopathologic structure of the original tumor thus recapitulating the heterogeneity observed in patients [56, 78, 128, 129]. This characteristic makes them a better tool compared with cells lines for studying drug efficacy and development: in fact, substantial limitations have been observed with conventional cell line–derived xenograft models for drug screening and evaluation of pre-clinical efficacy of drugs due to the loss of hallmarks, such as genetic and epigenetic alterations, resulting in minimal resemblance to the parental tumors [128, 130].

PDX mice are created via subcutaneous or orthotopic implantation into an immunodeficient mouse, and in contrast to cell line–derived xenografts, they are not propagated on plastic. There is wide variation in engraftment rates and time-to-engraftment among different cancers that may be impacted by the method used to implant the tumor (i.e., subcutaneous, orthotopic, or kidney capsule) and mouse strains [78, 128, 131, 132].

PDX are commonly used worldwide in pre-clinical trials for the development of anti-cancer drugs to support and validate the translation into clinical trials. In fact, many global PDX repositories have been generated and are currently available for pre-clinical research (https://www.europdx.eu/; https://www.pdxfinder.org/; https://www.crownbio.com/; https://championsoncology.com/; https://www.jax.org/; http://www.pdx.dnalink.com/index) [133].

The most recent application of these fine pre-clinical models have been “co-clinical trials”, where PDX, also known as “avatar” or “mirror” models, are generated using specimens derived from patients participating in the clinical trials and pre-clinical studies which are run in parallel, in real time, to the human trials. This approach has been effectively used in the past few years as a model for personalized medicine because they are able to longitudinally predict, with high accuracy, drug response, or resistance before these events can be observed in the donor patient [134–140].

#### Challenges and limitations of PDX models

The PDX mouse model is frequently cited as being highly representative of human tumors in terms of heterogeneity, clonal evolution, and response to treatment [81, 131, 141–145]. Recently, groups with wide expertise in the model have sought to assess the validity of PDX models in recapitulating different types of cancers and have highlighted many of the limitations and challenges of PDX models [128, 131, 146]. All groups working with PDX models argue that more studies should incorporate standardized validation tools to improve the reproducibility and to increase success rates of translational studies [72, 78, 79, 131, 132, 146].

Among some of the main challenges is the gradual replacement of human stroma with mouse stroma. In fact, after few passages, tumor-associated stroma gets replaced with murine-derived ECM (extracellular matrix) and fibroblasts, causing changes in the paracrine regulation of the tumor that might interfere with drug distribution and effectiveness [147].

Another challenge is the route of implantation. There are open questions surrounding the most favorable route of administration indicating the need for validation studies to address the optimal implantation site. Although orthotopic models seem to better mimic metastatic cancer models, subcutaneous administration is more commonly used because it is easier to assess drug efficacy [138].

The time course of engraftment is also a formidable challenge and is a limiting factor in the use of PDX models for co-clinical trials. Some models take 4–8 months to establish and this is more than what patients can wait to start treatment. In order to overcome this issue, some groups are switching to the use of organoids models to evaluate potential treatment sensitivity [24, 25].

Lastly, in order to establish standard PDX models, a key requirement is that the mice cannot have an intact immune system. This has impeded the use of PDX mice in studies assessing immune checkpoint–blocking agents [148, 149]. This is also driven, in part, by gradual replacement of engrafted stromal cells (and immune cells found in the tumor) with mouse cells leading to a more murine-like tumor microenvironment [131]. For these reasons, the development of humanized PDX models where the immune system is reconstituted in the PDX-implanted mouse represents a potential advancement for researchers [149, 150].

Nevertheless, despite these challenges, PDX models are considered among the most robust and clinically relevant models for drug screening and drug discovery. In fact, since 2016, the National Cancer Institute (NCI) has stopped using the NCI-60 panel (containing 60 human cancer cell lines) and switched to PDX models for anti-cancer drug screening [56, 78, 79, 82, 128, 133, 137, 138, 141, 151].

#### Metastasis

As previously mentioned, PDX models present limitations for studying metastasis, particularly if subcutaneous transplantation is used [55, 66, 131, 152]. The combination of the absence of an intact immune system and mouse stromal environment can influence disease progression and metastasis [66, 153]. Recently, Sprouffske et al. assessed this issue and used a bioinformatics approach to investigate the genetic heterogeneity during breast cancer metastasis [152]. Using WGS, they discovered that the mouse stromal environment can confound interpretation of intra-tumor heterogeneity and that the method of developing the PDX metastasis models can influence genetic changes in occurring during metastasis [152]. For example, tail vein injection of breast cancer PDX metastasis models exhibits a loss of heterozygosity compared to PDX mice with orthotopically transplanted breast cancer tumors that develop metastasis [152]. Thus, although a liver metastasis–derived PDX will better recapitulate human tumors, both cell line–derived and PDX *in vivo* subcutaneous models are limited in their ability to simulate metastasis, and more studies are required to address this unmet need.

#### Immune-tumor interactions

As noted several times, in addition to limitations in modeling metastasis, PDX models are immunocompromised as they are propagated in mice that lack a fully adaptive immune system. Thus, the influence of immune cells on tumor growth and response to treatment is poorly assessable in this model. However, there are several groups currently working to create mouse models with partial immune systems, primarily human T cells, to evaluate therapeutics used in the clinic, each which comes with challenges that will be discussed [149].

Several strains of mice can be used to generate PDX models. Traditional PDX models use athymic nude mice or severe combined immunodeficiency (SCID) mice [89]. Athymic nude mice lack T cells but still retain B cells as well as many elements of their innate immune response including NK cells and neutrophils [89]. Athymic mice are suitable hosts for human cancer cell lines, but NSG are more suitable for hosting human primary tumors [89, 154]. SCID mice, on the other hand, have genetically impaired VDJ recombination leading to disruption in T and B cell development with commensurate deficiency in these critical adaptive immune system cells and therefore cannot be used to evaluate anti-PD1 or anti-CTLA4 immunotherapy agents [89, 90]. In response to this, several groups are developing humanized PDX models reconstituted with human immune systems using novel approaches. These include reconstituting immunodeficient mice with mature immune cells (i.e., PBMCs or tumor-infiltrating lymphocytes) prior to transplanting patient tumor tissue containing human stromal cells along with any tumor-infiltrating human immune cells, while other groups have begun developing “humanized mice” reconstituted with human CD34^+^ hematopoietic stems cells (HSCs) following sub-lethal irradiation in immunodeficient mice, thereby repopulating them with a largely human immune system that includes B and T lymphocytes as well as myeloid cells [55, 149, 150, 155–159].

The benefit of the PMBC method is the ability to transplant patient-matched immune cells from the blood into mice prior to tumor challenge thereby limiting antitumor immune effects derived from allogeneic responses due to HLA mismatching [131]. Furthermore, it was recently demonstrated that tumor-specific T cells can be found in the circulation of cancer patients and may be important for responses to PD1 blockade [160]. Previous studies have demonstrated improved overall engraftment that results in high chimerism of human lymphocytes using mice with deficiencies in both their adaptive and innate immune systems as recipients [161].

Yet, there are drawbacks. First, not all immune cells engraft equally as many murine-derived cytokines do not cross-react with human receptors. This is particularly true for myeloid cells and is currently being addressed by various methods that include exogenous injection of growth factors and expression of human FLT3L [162]. Furthermore, the cells that do engraft undergo important phenotypic changes following repopulation of lymphogenic hosts that also further skew immune composition and activation. Most importantly, this xenograft system is limited by the narrow window of time for therapeutic studies as mice invariably succumb as a result of elicitation of profound xenograft-versus-host disease (xeno-GVHD) [131]. Ongoing studies are currently working to overcome this by using MHC-null NOG mice as recipients that have successfully been used for immunotherapy studies [163].

Another approach to overcoming severe xenograft versus host responses is transferring human CD34^+^ HSCs into irradiated, immunodeficient mice. As opposed to models with PBMCs containing mature T cells, the lymphocytes in the HSC transplant model develop in the murine host and therefore do not attack the host as a result of negative selection of thymocytes against murine MHC-peptide complexes [164]. In this system, human-derived thymocytes develop in the hosts’ thymus that are dominated by murine MHC-peptide complexes on thymic epithelial cells (TECs), which are important for both positive and negative selection [165]. Although human MHC-peptide complexes derived from donor immune cells are also present in the thymus, it is currently unknown what fraction of mature T cells are restricted to human versus mouse MHC peptides. Thus, counterintuitively, this model may initiate xenograft responses against human tumors upon challenge due to the absence of human MHC and peptides during negative selection. One way groups are attempting to overcome this issue is by introducing HLA genes in MHC-null mice that result in HLA-restricted T cells, albeit primarily HLA presenting murine rather than human peptides [166, 167]. Currently, this approach is limited to a specific HLA gene and cannot match the complete HLA haplotype of the patients’ tumor. Lastly, other groups have also demonstrated that implanting human thymuses in HSC-reconstituted mice helps select for HLA-restricted T cells and ensure proper negative selection against human peptides [168]. In summary, there are well-designed experiments ongoing to develop and refine PDX models for use in testing immunotherapy, but it is unlikely that any of them will be feasible for large scale use or high throughput screening.

Greater emphasis has recently been devoted to the establishment of humanized PDX models in order to investigate the tumor and immune compartment effects of treatment as well as the interplay between these two systems. Indeed, PDX models show convincing evidence for their ability to model drug response in multiple human cancer types including breast cancer, ovarian cancer, SCLC, adrenal, and CRC [56, 149, 150, 169]. There are a number of humanized mouse models with human-derived xenograft tumors as well as human immune systems that are currently in development and in use for drug screening and modeling of various oncologic diseases [170]. These models include bladder cancer humanized mouse models using NSG mice injected with CD34^+^ hematopoietic cells, breast cancer models created with NSG mice intrahepatically engrafted with breast carcinoma cell lines and engrafted with functional human immune systems, and CRC models via Rag2^*−/−*^*y*c^*−*/*−*^ mice injected with human PBMC’s and subcutaneously engrafted on the flank with CRC cell line HT-29 [171, 172]. These systems are promising models of immune system and tumor interactions, as with the humanized breast cancer model created by Wege et al., where human immune cells are able to traffic and infiltrate the microenvironment and enable human tumor-immune system interactions to be studied [171]. In summary, humanized oncological models may address vital questions on tumor-immune system interactions, mechanisms of tumor escape, and therapeutic potential of immune modulation, and may be of significant importance in predicting response to immunotherapy such as checkpoint inhibition in the future [171, 173].

#### Drug discovery and drug screening

To date, perhaps the most significant role for PDX in translational cancer research is in assessing drug response and translating *in vivo* drug screening data to the clinic [78, 128, 131]. A recent systematic review of retrospective studies correlated patient response in multiple oncologic indications with PDX response *in vivo* for cancer types including breast, ovarian, and small-cell lung cancer [169]. In support of the predictive capabilities of PDX models, they note a study that used a panel of seven human breast cancer patient-derived orthotopic xenografts (PDOXs) to predict patients’ responses to the chemotherapeutics docetaxel and 5-FU given in combination therapy with the monoclonal antibody trastuzumab, revealing an overall concordance in five of seven patients and corresponding PDOX models [169]. In the case of ovarian cancer, another study found that 19 of 21 PDXs exhibited congruent results of sensitivity or resistance with patients in retrospective studies with cisplatin treatment [169]. Lastly, in SCLC, strong correlations between the response rate to chemotherapy with cisplatin and etoposide combination treatment between SCLC patients and PDXs were demonstrated in seven of nine patient PDOX pairs [169]. While this systematic review highlights the potential role of PDX models in predicting tumor drug responses in certain cancer types such as breast cancer, ovarian cancer, and SCLC, primary research utilizing larger cohorts in other cancer types has additionally yielded interesting results. For example, Bertotti et al. produced a large cohort of patient-derived xenografts from 85 patients with metastatic colorectal cancer and characterized response to cetuximab (an anti-EGFR antibody) in correlation to patients in clinic [56]. In this panel of CRC patients and corresponding PDXs, all 85 were concordant regarding treatment response or resistance to cetuximab treatment. For treatment with cetuximab, the response rate (11%), disease stabilization (30%), or progression (59%) was in line with the data reported in the prospective analysis of the patients. Importantly, metastatic CRC xenografts retained the morphologic characteristics of the corresponding patient’s tumor, and serial mouse passaging did not substantially alter the genetic makeup of tumors as it related to copy number changes and hotspot oncogenic mutations. This indicates that in addition to being of benefit for prediction of drug response in CRC, PDX mouse models also retain key features of human tumors despite passaging and mouse stromal invasion [56]. However, the validity of PDX models as predictive tools in translational research has been questioned suggesting more validation tools are needed as part of PDX studies [146].

In modelling of drug pharmacokinetic and dynamic properties, PDX models have demonstrated promise in some systems while exhibiting limitations in others. Wong et al. carried out a retrospective PK/PD analysis of clinical response data from 8 well-characterized cytotoxic agents including 5-FU and docetaxel in comparison with response and PK/PD parameters in corresponding xenograft models with these same agents [174]. It was observed that regimens of docetaxel that were varied by dose and cycle duration in metastatic breast cancer models, such as the Cal51x1.1s PDX exhibited similar parameters of tumor growth inhibition and overall response to those documented in the clinical setting. Likewise, modelling of PK/PD parameters for treatment of CRC with 5-FU demonstrated that continuous infusion exhibited superior performance in comparison with a 5-day regimen in the Colo205 PDX model and was representative of clinical response in both cases [174]. Meanwhile, previous studies by Combest et al. concluded that genetically engineered transgenic mouse models may more closely recapitulate drug pharmacokinetic properties in comparison with PDX models in the setting of melanoma [126]. The limited pharmacokinetic data that exists for direct comparison of PDX and GEMM systems demonstrate that GEMM models may be superior in accurately modelling drug PK parameters in some cancer types [126].

## Conclusion

Each of the tools discussed here plays important roles in unraveling tumorigenesis, identifying drug targets, and ascertaining drug efficacy (Fig. 1). Two-dimensional cell culture still has a prominent role in cancer research and a long history of generating valuable results on underlying genetic changes in cancer that has led to the discovery of drug targets and therapeutic agents. Indeed, cell culture is a simple and cheap means to screen compounds and candidate drugs prior to more elaborate and predictive *in vivo* models. However, on its own, cell culture is subject to such profound shifts in gene expression with prolonged culture that its translational value is limited. The principle uses of cell culture are for preliminary drug screening/drug discovery (Fig. 1). Although there has been work on cancer immunology using cell culture, there are better systems available as noted. Likewise, cell culture is not ideally suited for the study of metastasis, and while there are migration, invasion, and metastasis assays, they lack the robustness of mouse models.

Organoids are a more elaborate form of cell culture that can be used to study tumor heterogeneity (Fig. 1). Organoids also appear to be effective and predictive platforms for drug screening and discovery, more so than cell culture but less than some mouse models (Fig. 1). Similar to cell culture, organoids are not well suited for studying cancer immune function or metastasis.

Comparison of *in vivo* mouse model systems including xenograft, syngeneic, GEMMs, and PDX models reveals that there are unique benefits and limitations to each (Fig. 1). Of these, cell line–derived xenograft mouse models appear to be the least valuable for studying tumor heterogeneity, tumor evolution, and immune-tumor interactions, but have demonstrated some success in studying metastasis and drug action (Fig. 1). While syngeneic models offer a competent immune system, as discussed, particular care needs to be taken when choosing the murine tumor cell line used. Significant differences in genetic composition and immunologic profile have been demonstrated in this model within the same cancer type, and drug response results have been shown to differ between syngeneic models and human subjects for multiple indications [63, 65, 71]. Further concern regarding this model system is the lack of similar stromal and immune compartments in the tumor microenvironment following injection of tumor cells [89]. Although not without limitations, GEMMs appear to be a reasonable option for studying tumor heterogeneity, tumor evolution, metastasis, and to a lesser degree, immune-tumor interactions (with a mouse immune system). Although GEMMs also have predictive value in modelling drug response and PK parameters, they are still mouse tumors [123, 126]. PDX mouse models remain the strongest *in vivo* model for predicting drug response in patients [128] (Fig, 1). However, limited data for direct comparison of PDX and GEMM systems regarding PK modelling has demonstrated PDX tumors may be inferior in accurately recapitulating drug PK parameters in some cancer types [126, 175], and further studies are needed. As noted, the lack of an immune system hampers the use of PDX mice in studying immune-tumor interactions or the effects of immunotherapy agents. However, the advent of humanized PDX mice has helped to fill this gap (Fig. 1).

Ultimately though, it is through the use of combinations of models that the ideal system may come closest to realization. Combining translational models is now frequently employed to leverage the advantages each system has to offer. The work described earlier by Lu et al., for example, typifies this translational combinatorial approach. Here, they describe how both syngeneic mouse models, which spontaneously develop tumors that aggressively metastasize to the lungs, were used in tandem with NSG mice transplanted with these tumors for drug screening [116]. Furthermore, coupling *in vivo* models with single-cell analysis technology and computational assays also stands to extend the resolution of data gleamed from any one technique.

Finally, there are a number of promising approaches being developed that extend beyond the scope of the current review and into clinical cancer diagnosis, but merit consideration given their potential use in pre-clinical cancer research. Advancements in imaging and spectroscopy have led to refinement of technology such as Raman spectroscopy and mass spectrometry for use in cancer detection and diagnosis [176, 177]. Thus, it is conceivable that these technologies could be used in tandem with mouse models, such as PDX mice, to detect metastatic tumor formation and circulating tumor cells thereby extending the role of these models in cancer drug development.
